# Searching for homozygous haplotype deficiency in Manech Tête Rousse dairy sheep revealed a nonsense variant in the *MMUT* gene affecting newborn lamb viability

**DOI:** 10.1186/s12711-024-00886-7

**Published:** 2024-02-29

**Authors:** Maxime Ben Braiek, Carole Moreno-Romieux, Céline André, Jean-Michel Astruc, Philippe Bardou, Arnaud Bordes, Frédéric Debat, Francis Fidelle, Itsasne Granado-Tajada, Chris Hozé, Florence Plisson-Petit, François Rivemale, Julien Sarry, Némuel Tadi, Florent Woloszyn, Stéphane Fabre

**Affiliations:** 1 GenPhySE, Université de Toulouse, INRAE, ENVT, 31326 Castanet-Tolosan, France; 2 CDEO, Quartier Ahetzia, 64130 Ordiarp, France; 3 Institut de l’Elevage, 24 Chemin de Borde-Rouge, 31321 Castanet-Tolosan, France; 4 INRAE, 31326 Castanet-Tolosan, France; 5 Department of Animal Production, NEIKER-BRTA Basque Institute of Agricultural Research and Development, Agrifood Campus of Arkaute s/n, 01080 Arkaute, Spain; 6 Eliance, 149 Rue de Bercy, 75595 Paris, France; 7 GABI, Université Paris-Saclay, INRAE, AgroParisTech, 78350 Jouy-en-Josas, France

## Abstract

**Background:**

Recessive deleterious variants are known to segregate in livestock populations, as in humans, and some may be lethal in the homozygous state.

**Results:**

We used phased 50 k single nucleotide polymorphism (SNP) genotypes and pedigree data to scan the genome of 6845 Manech Tête Rousse dairy sheep to search for deficiency in homozygous haplotypes (DHH). Five Manech Tête Rousse deficient homozygous haplotypes (MTRDHH1 to 5) were identified, with a homozygous deficiency ranging from 84 to 100%. These haplotypes are located on *Ovis aries* chromosome (OAR)1 (MTRDHH2 and 3), OAR10 (MTRDHH4), OAR13 (MTRDHH5), and OAR20 (MTRDHH1), and have carrier frequencies ranging from 7.8 to 16.6%. When comparing at-risk matings between DHH carriers to safe matings between non-carriers, two DHH (MTRDHH1 and 2) were linked with decreased insemination success and/or increased stillbirth incidence. We investigated the MTRDHH1 haplotype, which substantially increased stillbirth rate, and identified a single nucleotide variant (SNV) inducing a premature stop codon (p.Gln409*) in the *methylmalonyl-CoA mutase* (*MMUT*) gene by using a whole-genome sequencing approach. We generated homozygous lambs for the *MMUT* mutation by at-risk mating between heterozygous carriers, and most of them died within the first 24 h after birth without any obvious clinical symptoms. Reverse transcriptase-qPCR and western blotting on post-mortem liver and kidney biological samples showed a decreased expression of *MMUT* mRNA in the liver and absence of a full-length MMUT protein in the mutant homozygous lambs.

**Conclusions:**

We identified five homozygous deficient haplotypes that are likely to harbor five independent deleterious recessive variants in sheep. One of these was detected in the *MMUT* gene, which is associated with lamb lethality in the homozygous state. A specific management of these haplotypes/variants in the MTR dairy sheep selection program would help enhance the overall fertility and lamb survival.

**Supplementary Information:**

The online version contains supplementary material available at 10.1186/s12711-024-00886-7.

## Background

In livestock, genetic selection has largely improved production traits over the past decades, but in the last 10 years, the emergence of new technological tools has allowed the implementation of genomic selection, which further enhanced the genetic progress [1]. The availability of high-density single nucleotide polymorphism (SNP) chips and improved reference genomes and gene annotations have allowed to fine-map genomic regions and identify causal variants associated with production traits [1–4]. While successful, selection on these traits is accompanied by a decline in fertility as observed in dairy cattle [5]. Although the environment explains a large part of the reproductive performance in ruminants, genetic studies have made it possible to correct this trend and improve fertility although its heritability is lower than 0.05 [6–8]. These studies have shown that Mendelian monogenic disorders are one of the causes of fertility failure [9].

Today, two main approaches are widely used to identify recessive deleterious variants. The first approach is a classical forward genetic screen, which is based on a case–control analysis and genome-wide association studies [10]. This method requires distinctive phenotypes to distinguish between affected and unaffected animals, and biological samples from affected animals. Then a homozygosity mapping approach can be used to identify homozygous regions in affected animals that are likely to harbor the causal variant. This can be further confirmed by whole-genome sequencing (WGS) data [11, 12]. However, this approach has some limitations when biological samples and descriptive phenotypes are not available. To overcome these, a second approach of reverse genetics was developed to specifically identify recessive lethal variants. This strategy, initially developed by VanRaden et al. [13] in cattle, exploits the availability of large numbers of genotyped animals from genomic selection datasets to detect haplotypes showing deficit in homozygous animals, with a significant deviation from Hardy–Weinberg equilibrium. Initially, this method was developed to detect embryonic lethal variants, but it can also be used to fine-map deleterious variants that lead to neonatal or juvenile lethality and morphological disorders. Recently, we applied this approach for the first time in sheep, identifying eight independent deficient homozygous haplotypes in the Lacaune dairy breed [14]. Then, we focused on the Lacaune deficient homozygous haplotype 6 (LDHH6, OMIA 002342–9940), and identified a nonsense variant in the *CCDC65* gene that causes juvenile mortality associated with respiratory distress when it is in the homozygous state [15].

In this study, we used dense genotype data to search for lethal variants in Manech Tête Rousse (MTR) dairy sheep. This breed is raised in the French Basque Country and is the second most important breed by population size in France (~ 450,000 ewes) [16]. Genetic variability is well controlled in the MTR breed, with an increase of + 0.4% inbreeding per generation in the 1999–2009 period. The effective population size ranges from 110 to 200, depending on the estimation method used [16–18]. Genomic selection was implemented in MTR in 2017, as for other dairy sheep breeds in France [19]. The objective was to identify deficient homozygous haplotypes by applying a reverse genetic screen using the large amount of genotyping data available in MTR dairy sheep and to test the hypothesis of negative impacts on fertility traits in at-risk matings. We focused on a specific region to pinpoint the causal variant from WGS data and arrange at-risk matings between carriers to establish the associated phenotype.

## Methods

### Animal and genotyping data

The complete dataset is composed of 6845 genotyped Manech Tête Rousse animals (82% males and 18% females) born between 1993 and 2021 [for data description (see Additional file 1: Fig. S1)]. Genotyping was performed at the Labogena facility (http://www.labogena.fr/) within the framework of the implementation of genomic selection in French dairy sheep [19]. Both low-density (LD) 15 k SNP chips (SheepLD; n = 2956) and medium-density (MD) 50 k SNP chips (Ovine SNP50 BeadChip; n = 3889) were purchased from Illumina Inc. (San Diego, USA) (Table 1). Pedigree information was extracted from the official national database SIEOL (*Système d'Information en Elevage Ovin Laitier*, France).

**Table 1:** Description of genotyped animals

Year of birth	< 2017	≥ 2017		Total
Background	Research programs	Genomic selection and research programs		
Number of animals	2533 rams	3077 rams		6845
	692 ewes	543 ewes		
SNP chip	MD	LD	MD	LD (n = 2956)
	(n = 3225)	(n = 2956)	(n = 664)	MD (n = 3889)
Genotyping age	> 12 months	~ 15 days	8–12 months	

*MD* medium density (50 k), *LD* low density (15 k)

### Genotype quality control, imputation and phasing

Quality control for each SNP was carried out following the French genomic evaluation pipeline based on (*i*) a call frequency > 97%, (*ii*) a minor allele frequency > 1%, and (*iii*) no deviation from Hardy–Weinberg equilibrium $\text{(P} \, {>} \, {10}^{-5}$). Genotypes were phased and imputed from LD to MD using *FImpute3* [20]. The accuracy of the LD to MD imputation in MTR was previously assessed and resulted in a concordance rate per animal of 98.86%, a concordance rate per SNP of 98.95% and a squared Pearson’s correlation coefficient of 93.57% between imputed and observed SNP genotypes [21]. After quality control, the 38,523 remaining autosomal SNPs were mapped to the *Ovis aries* genome assembly Oar v3.1 (current version used in the French genomic evaluation) [22]. These SNPs were also located on the genome assembly Oar_rambouillet_v1.0 (GCF_002742125.1). Genomic coordinates for both versions of the sheep genome are available at 10.6084/m9.figshare.8424935.v2 (https://sheephapmap.org).

### Detection of homozygous haplotype deficiency

To identify deficient homozygous haplotypes, we screened the genome of 5271 genotyped animals that belonged to trios (77 offspring had both parents genotyped, and 4799 offspring had both sire and maternal grandsire genotyped). Briefly and as previously described [14], the method consists in (*i*) screening the genome using a sliding window of 20 SNPs, (*ii*) selecting all 20-SNP haplotypes that had a frequency higher than 1% on the maternal phase, (*iii*) comparing the observed number ${N}_{{\text{Obs}}}(k)$ to the expected number ${N}_{{\text{Exp}}}(k)$ of homozygous offspring for each haplotype $k$ using within-trios transmission probability and further considered the haplotypes with $\text{P-Poisson} \, {<} \, \text{1.9}\times{10}^{-4}$, and (*iv*) retaining deficit between 75 and 100% defined as ${(N}_{Exp}\left(k\right)-{N}_{Obs}\left(k\right))/{N}_{Exp}\left(k\right).$ Finally, we clustered consecutive windows with the same deficit to define larger regions called "Manech Tête Rousse deficient homozygous haplotypes" (MTRDHH). Then, we determined the status (homozygous non-carriers, heterozygous, or homozygous carriers) of each MTRDHH region for all 6845 genotyped animals. Linkage disequilibrium was estimated between two MTRDHH regions located on the same chromosome using the r^2^ coefficient measure [14].

### Analysis of fertility traits

Mating records of MTR sheep between 2006 and 2019 were obtained from the national database SIEOL. We analyzed only artificial insemination success (AIS) and stillbirth rate (SBR) records from matings where both the sire and maternal grand sire were genotyped (i.e., had a known status at each MTRDHH). AIS was coded as "1" for success and "0" for failure based on lambing date, according to the gestation length starting from the day of artificial insemination (AI) (151 ± 7 days; n = 330,844 mating records). SBR was determined only in the AIS success group, and coded as "1" if there was at least one stillbirth in the litter (stillbirth records also included lamb deaths within the first 24 h after birth) or "0" if all lambs were born and still alive 24 h after birth (n = 201,637 mating records). We considered a mating between a carrier ram and a ewe from a carrier sire to be an “at-risk mating”. We considered other combinations to be “safe matings”: (*i*) non-carrier ram × ewe from a non-carrier sire, (*ii*) non-carrier ram × ewe from a carrier sire, and (*iii*) carrier ram × ewe from a non-carrier sire. A logistic threshold binary model with a logit link function was used to compare AIS and SBR between at-risk and safe matings (lsmeans estimate), using the GLIMMIX procedure in the SAS software (version 9.4; SAS Institute Inc., Cary, NC). The fixed effects for AIS and SBR were mating type (safe or at-risk), season of AI (spring or summer), and lactation number (L1, L2, L3 and L4 +). For SBR only, prolificacy of the ewe (1, 2, 3 + lambs/litter) was added as a fixed effect. For AIS and SBR, the random effect was the herd × year interaction (n = 313 herds between 2006 and 2019). Traits were considered to differ significantly when the mating type fixed effect had a P-value lower than 1.0 × 10^–2^ after Bonferroni correction for multiple testing with a level of significance α at 5%. This threshold was obtained by dividing the level of significance α by the number of tests corresponding to the number of independent haplotypes (n = 5).

### Analysis of milk parameters and total merit genomic index (ISOLg)

Daughter yield deviations (DYD) for milk traits from genotyped sires with known status at each MTRDHH were computed from official genetic evaluations (GenEval, Jouy-en-Josas, France). The DYD corresponds to the average performance of the daughters of each sire, corrected for environmental effects and the average genetic value of the dam [19]. The six traits studied were milk yield (MY), fat (FC) and protein (PC) contents, fat (FY = MY × FC) and protein (PY = MY × PC) yields, and lactation somatic cell score (LSCS) as described [14]. To compare all the traits on the same scale, each DYD was divided by its genetic standard deviation to obtain standardized DYD (sDYD). For all milk production traits, positive values indicate an improvement of the selected traits, whereas for the lactation somatic cell score, a positive value indicates a deterioration of udder health in the progeny of heterozygous rams. Only genotyped rams with records from at least 20 daughters were included in the analysis to obtain sufficiently accurate DYD values (n ~ 2570 rams). Each trait was tested by variance analysis comparing MTRDHH haplotype carrier and non-carrier rams using the GLM procedure in the SAS software (version 9.4; SAS Institute Inc., Cary, NC). The fixed effects were the genetic status (carrier or non-carrier) and year of birth (2000 to 2016) to correct for annual genetic gain. Traits were considered to differ significantly when the genetic status had a P-value lower than 1.0 × 10^–2^ after Bonferroni correction for multiple testing with a level of significance α at 5%. This threshold was obtained as explained above for fertility traits (n = 5 independent haplotypes).

The total merit genomic index in dairy sheep (called “ISOLg”, *Index Synthétique des Ovins Laitiers*) was extracted from the official genomic evaluation (GenEval, Jouy-en-Josas, France) for all the 714 genomic candidate lambs born in 2021. ISOLg is determined by a combination of four selected traits: MY, FC, PC and LSCS. ISOLg from heterozygous carrier and non-carrier lambs were compared for each MTRDHH region, with a Wilcoxon non-parametric test under the null hypothesis with a significance level of α = 5% using the “wilcox.test” function in the R software (version 4.1.3, R Core Team, 2022).

### Whole-genome sequencing data

Publicly available data of 100 ovine short-read Illumina HiSeq/NovaSeq whole-genome sequences (WGS) from 14 breeds, generated in various INRAE and Teagasc research projects, were used for variant calling. Of these, 22 WGS were obtained from MTR dairy sheep that were also genotyped with the MD SNP chip. A description of the different breeds and the accession numbers of the sequencing raw data are available (see Additional file 2: Table S1).

### WGS variant calling and filtering

Read mapping, variant calling and functional annotation were performed using the Nextflow v20.10.0 and Sarek v2.6.1 pipelines for the 100 short-read WGS as previously described [15]. The region of interest (MTRDHH1 region extended by 1 Mb on each side) was extracted using SnpSift Filter, part of the SnpEff toolbox [23]. Candidate variants were filtered based on the Pearson correlation between haplotype status (homozygous non-carriers, heterozygous and homozygous carriers encoded as 0, 1 and 2, respectively) and allele dosage for bi-allelic variants (also encoded 0, 1 and 2) using the geno–r2 command of VCFtools [24]. Only variants with a perfect correlation with haplotype status (r^2^ = 1) were retained.

### Specific variant genotyping assay

The SNV NC_040271.1: g.23,776,347G > A in the *methylmalonyl-coA mutase* (*MMUT*) gene was genotyped by PCR allele competitive extension (PACE) analysis with 15 ng of purified DNA using the PACE-IR 2 × Genotyping Master mix (3CR Bioscience) in the presence of 12 µM of a mix of extended allele specific forward primers and 30 µM of common reverse primers in a final volume of 10 μL [for primer sequences (see Additional file 3: Table S2)]. The touch-down PCR amplification conditions were 15 min at 94 °C for the hot-start activation, 10 cycles of 20 s at 94 °C, 54–62 °C for 60 s (decreasing by 0.8 °C per cycle), then 36 cycles of 20 s at 94 °C and 60 s at 54 °C performed on an ABI9700 thermocycler followed by a final point read of the fluorescence on an ABI QuantStudio 6 real-time PCR system and using the QuantStudio software 1.3 (Applied Biosystems).

The presence of the *MMUT* variant was checked in a DNA set of the 2021 cohort of 714 MTR male lambs that were candidates for genomic selection. The DNA was extracted by Labogena (Jouy-en-Josas, France), on behalf of the MTR breed industry. In addition, another DNA set included a diversity panel of 851 animals from 25 French sheep breeds [25] and three Spanish sheep breeds.

### Generation of homozygous lambs

Blood samples (3 mL) were first collected from 181 ewes, which were daughters of MTRDHH1 haplotype carrier sires, located on six private farms, by jugular vein puncture with the Venoject system containing EDTA (Terumo, Tokyo, Japan) and directly stored at -20 °C. Among these, 82 ewes were genotyped as heterozygous carriers of the *MMUT* variant and were selected to be inseminated with *MMUT* variant heterozygous rams (at-risk matings). Experiment 1 (n = 73 ewes) was performed on six private farms in Pays-Basque (France) and Experiment 2 (n = 9 ewes) was performed on the INRAE experimental farm of Langlade under the agreement number E31429001 (Pompertuzat, France, 10.17180/ftvh-×393). The experimental design is described in Additional file 4: Fig. S2. An ultrasound diagnosis of gestation was performed between 45 and 60 days after AI. Gestations were followed and each lamb was monitored form birth to weaning. Ear biopsies (1 mm^3^) from the 72 lambs born in both experiments were obtained with a tissue sampling unit (TSU, Allflex Europe, Vitré, France) and directly placed in the TSU storage buffer at 4 °C. Ear biopsies were placed twice consecutively in 180 μL of 50 mM NaOH, heated 10 min at 95 °C, neutralized with 20 μL of 1 M Tris–HCl, and then vortexed during 10 s. All neutralized samples were used for direct genotyping without DNA purification as described in [26]. In Experiment 2, all lambs were weighed at birth. Biological samples (plasma, urine, liver and kidney) were collected on animals as described in Additional file 4: Fig. S2 and frozen at − 80 °C until use.

### Immunoblot analysis

Frozen kidney and liver tissues were crushed in liquid nitrogen using a Mixer Mill during 30 s at 30 Hz (MM400, Retsch technology), and 12 mg of tissue powder were lysed with 500 µL of RIPA solution (Ref#R0278, Sigma-Aldrich). The protein extracts were centrifuged for 20 min at 16,000 g and 4 °C, and protein concentration in the supernatant was determined using the BCA protein assay Kit (Ref#K812-1000, Biovision). Each protein sample (45 µg) was denatured and reduced in Laemmli buffer (62.5 mM TRIS pH 6.8, 2% SDS; 10% glycerol) containing 5% ß-mercaptoethanol before SDS-PAGE on a 4–15% polyacrylamide gel (Bio-Rad). Proteins were transferred onto a nitrocellulose membrane blocked with AdvanBlock-Chemi blocking solution (Advansta) during 1 h at room temperature. After washing in PBS-0.1% Tween 20, the membrane was incubated overnight at 4 °C with a rabbit polyclonal anti-MMUT primary antibody at 1/1000 in blocking solution (MUT Rabbit pAb, Ref#A3969, ABclonal). Revelation of the primary antibodies (after washing) was performed by incubation with goat anti-rabbit Horseradish peroxidase-conjugated secondary antibody (Ref#A0545, Sigma-Aldrick) at 1/10000 ratio in blocking solution for 1 h at room temperature, followed by enhanced chemiluminescence detection (WesternBright Quantum HRP substrate, Advansta) on a ChemiDoc Touch low-light camera (Bio-Rad) in automatic mode.

### RNA extraction, reverse transcription and quantitative PCR

Total RNA was extracted from 80 mg of frozen kidney (n = 15) and liver tissue powders (n = 15) in 1 mL Trizol reagent (Invitrogen, #Ref 15596-018) and isolated using the Nucleospin® RNA II kit (Macherey–Nagel, #Ref 740955.50) according to the manufacturer’s protocol and including DNAseI digestion treatment. The RNAs were quantified by spectrophotometry (NanoDrop® ND-8000 spectrophotometer, ThermoFischer) and stored at − 80 °C. Reverse transcription was carried out on 1 µg of total RNA in solution with anchored oligo(dT) T22V (1 µL at 10 μM), random oligo-dN9 (1 µL at 10 μM) and dNTPs (2 µL at 10 mM) in a reaction volume of 10 μL. This mixture was incubated at 65 °C for 5 min in an ABI2700 thermocycler (Applied Biosystems), and then ramped down to 4 °C. A second reaction mixture (8 μL/reaction) containing the reaction buffer (5 µL of First strand Buffer 5X, Invitrogen, France), DTT (Dithiothreitol, 1 µL at 0.1 M), Rnasine (1 µL, 40 units/µL, Promega, France) and Superscript II reverse transcriptase (1 µL, 200 units/µL, Invitrogen, France) was added to the denatured RNA solution (final volume reaction of 18 μL), and then incubated for 50 min at 42 °C and placed for 15 min at 70 °C. The complementary DNA (cDNA) solution obtained was directly diluted at a 1:5 ratio and stored at − 20 °C. For each pair of primers, amplification efficiency was evaluated by $E={e}^{-1/\alpha }$ where α is the slope of a linear curve obtained from cDNA serial dilution (1:5 to 1:80) and corresponding Ct (cycle threshold) values. Quantitative PCR (qPCR) was performed using 3 µL of cDNA at 1:20 ratio, 5 µL of SYBR Green real-time PCR Master Mix 2X (Applied Biosystems) and 2 µL of primers at 3 µM in a total reaction volume of 10 µL on a QuantStudio 6 Flex Real-Time PCR system (ThermoFisher). Each sample was tested in duplicate. RNA transcript abundance was quantified using the $\Delta \Delta Ct$ method corrected by four reference genes (*GAPDH*, *YWHAZ, RPL19* and *SDHA*) and a calibrator sample. Primers were designed using the Beacon Designer™ 8 (Premier Biosoft). The list of qPCR primer sequences, amplification length and amplification efficiency used is in Additional file 3: Table S2.

### Methylmalonic acid dosage assay

The quantification of methylmalonic acid (MLA) was performed on 11 urine and 15 plasma samples (100 µL), collected from lambs, using the Sheep MLA Elisa kit (MyBioSource, Ref# MBS7266308) following the manufacturer’s protocol. The incubation phase with the specific antibody was performed overnight at 4 °C. The optical density at 450 nm was determined using a GloMax®-Multi Detection System (Promega).

## Results

### Identification of deficient homozygous haplotypes in Manech Tête Rousse dairy sheep

Using a reverse genetic screen strategy based on 5271 genotyped animals belonging to trios, we detected 150 highly significant deficient homozygous haplotypes of 20 SNPs (see Additional file 5: Table S3 and Additional file 6: Fig. S3). These 150 haplotypes were clustered to five larger independent haplotypes called “Manech Tête Rousse Deficient Homozygous Haplotype” (MTRDHH). Three haplotypes showed a total deficit in homozygous animals (MTRDHH1, 2 and 3), whereas two haplotypes, MTRDHH4 and 5, only showed a partial deficit (84% and 91%) with one and eight homozygous animals genotyped while 11 and 49 were expected, respectively (Table 2). The complete description of MTRDHH SNPs (SNP name, SNP allele and position on sheep reference genomes Oar_v3.1, Oar_Rambouillet_v1.0 and ARS-UI_Ramb_v2.0) is available (see Additional file 5: Table S4). The different MTRDHH regions were located on *Ovis aries* chromosome (OAR)20 (MTRDHH1), OAR1 (MTRDHH2 and 3), OAR10 (MTRDHH4) and OAR13 (MTRDHH5), and their length ranged from 1.1 to 4.6 Mb on Oar_rambouillet_v1.0 genome assembly. The observed frequencies of heterozygous carriers ranged from 7.8 to 16.6%. MTRDHH2 and MTRDHH3 haplotypes (both located on OAR1) were not in linkage disequilibrium. Consequently, the five MTRDHH haplotypes identified are likely to harbor five independent variants causing the observed homozygous deficiency.

**Table 2:** List of Manech Tête Rousse deficient homozygous haplotypes

Haplotype	OAR	^a^Number of markers	^b^Position (Mb)	^c^Heterozygous carrier frequency (%)	Number of homozygotes			
					Exp.	Obs.	Deficit (%)	Poisson P-value
MTRDHH1	20	32	23.0–25.0	9.7	13	0	100	${2.9}\times{10}^{-6}$
MTRDHH2	1	66	251.9–256.4	8.7	10	0	100	${3.8}\times{10}^{-5}$
MTRDHH3	1	39	103.8–106.6	7.8	9	0	100	${9.6}\times{10}^{-5}$
MTRDHH4	10	26	30.5–31.5	8.7	11	1	91	${1.5}\times{10}^{-4}$
MTRDHH5	13	29	64.3–67.2	16.6	49	8	84	${5.3}\times{10}^{-13}$

*OAR* *Ovis aries* chromosome

*Exp* Expected

*Obs* Observed

^a^Details on the MTRDHH haplotypes and SNP composition are in Additional file 5: Table S4

^b^Position on the ovine genome assembly Oar_rambouillet_v1.0

^c^Frequency of carriers in the entire genotyped population (n = 6845)

### Impact of MTRDHH haplotypes on fertility traits

**Fig. 1:**
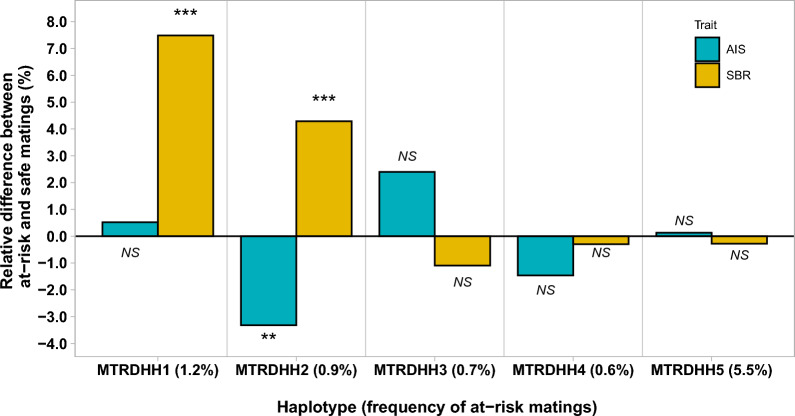
Effects of MTRDHH haplotypes on artificial insemination success (AIS) and stillbirth rate (SBR) between at-risk and safe matings. The frequency of at-risk matings for each MTRDHH haplotype is shown in parentheses. Significant effects were indicated by the corrected P-value for multiple tests, with a threshold set at α = 0.1% (**), 0.01% (***). NS indicates not significant

To identify a potential lethal effect of the five MTRDHH haplotypes, two fertility traits were analyzed: artificial insemination success (AIS), a proxy for embryonic loss (330,844 matings) and stillbirth rate (SBR) associated with perinatal lethality (201,637 matings) (Fig. 1). The average AIS of the population was 60.9%. When comparing at-risk and safe matings, only the MTRDHH2 haplotype showed a significant decrease of – 3.3% in AIS $\text{(P} \, {=}{ \, {3.5\times10}}^{-4}$) in at-risk matings.

The average SBR of the population was 7.5%. As described in Fig. 1, the MTRDHH1 and 2 haplotypes showed a significant increase in SBR with + 7.5% $\text{(P} \, {=} \, {4.0\times10}^{-24}$) and + 4.3% $\text{(P} \, {=} \, {1.3\times10}^{-6}$), respectively, in at-risk matings compared to safe matings. The three other haplotypes showed no significant impact on the fertility traits studied.

### Pleiotropic effects of MTRDHH haplotypes on milk production traits

**Fig. 2:**
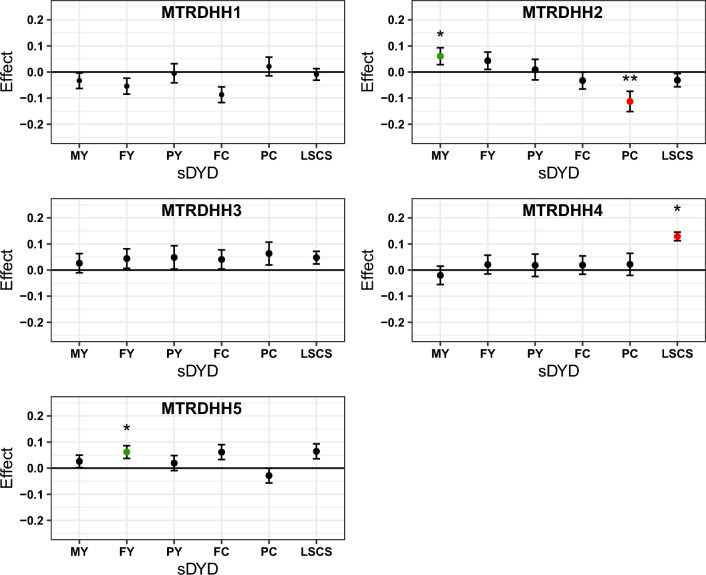
sDYD relative difference between heterozygous and non‑carrier rams for six traits under selection. *MY* milk yield, *FY* fat yield, *PY* protein yield, *FC* fat content, *PC* protein content, *LSCS* lactation somatic cell score, *sDYD* standardized daughter yield deviation (DYD divided by genetic standard deviation). sDYD relative difference values were obtained from lsmeans estimates according to mating class. Significant effects were indicated by the corrected P-value for multiple tests, with a threshold set at α = 5% (*), 1% (**). Error bars indicate standard errors. Significant favorable effects of heterozygous are in green, while significant unfavorable effects are in red

Six dairy traits are routinely included in genomic evaluation of the French dairy sheep. Standardized daughter yield deviation (sDYD) of five milk production traits (milk, fat and protein yields, and fat and protein contents) and lactation somatic cell score (a proxy for udder health) were compared between carrier and non-carrier rams for each of the five MTRDHH haplotypes (Fig. 2). Among the five haplotypes, three were associated with a significant effect on sDYD. Daughters of MTRDHH2 haplotype carrier rams showed a significant increase in milk production (sDYD + 0.06, ${\text{P}} \, {=}{ \, {6.5\times10}}^{-3}$) but a decrease in protein content (sDYD -0.11, ${\text{P}} \,{=}{ \, {4.7\times10}}^{-4}$). For the MTRDHH4 haplotype, there was a significant increase in lactation somatic cell score (sDYD 0.13, ${\text{P}} \, {=}{ \, {2.4\times10}}^{-3}$), and daughters of MTRDHH5 haplotype carrier rams showed a higher fat yield (sDYD 0.06, ${\text{P}} \, {=}{ \, {9.6\times10}}^{-3}$). In addition, the total merit genomic index (ISOLg), was extracted from each male lamb of the 2021 genomic selection cohort to estimate the impact of each MTRDHH on the genetic gain of the selected traits. No significant difference was observed on ISOLg between heterozygous carrier and non-carrier lambs for each MTRDHH (see Additional file 7: Fig. S4).

### Evolution of the MTRDHH haplotype frequencies in the population

**Fig. 3:**
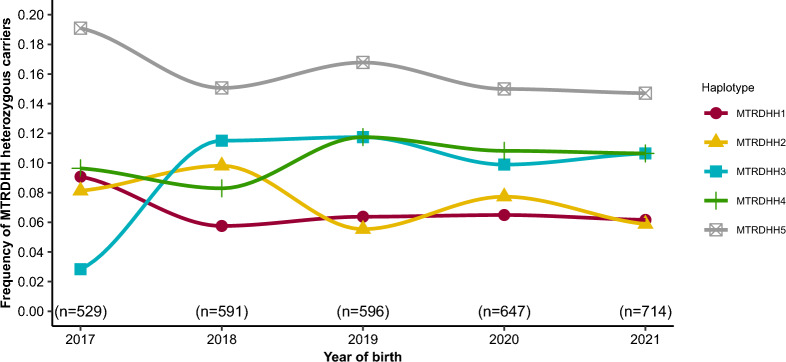
Evolution of the heterozygous carrier frequencies of MTRDHH haplotypes in Manech Tête Rousse male lambs between 2017 and 2021. The numbers (n =) refer to all candidates genotyped to enter the genomic selection scheme for each year

Since the implementation of genomic selection in 2017, all candidate rams from elite matings are genotyped on a low-density SNP chip when they are 7 days old. This reflects the genetic diversity that AI spreads in the selection scheme. As shown in Fig. 3, the frequencies of the MTRDHH haplotype heterozygous carriers were quite stable during the last five years and around 6.8, 7.4, 9.3, 10.2 and 16.1% for MTRDHH1, 2, 3, 4 and 5, respectively. Nevertheless, we can observe a significant increase in the frequency of the MTRDHH3 haplotype from 2.8 to 10.9% when comparing 2017 and 2018.

### Identification of a nonsense variant in the *MMUT* gene associated with the MTRDHH1 haplotype

**Fig. 4:**
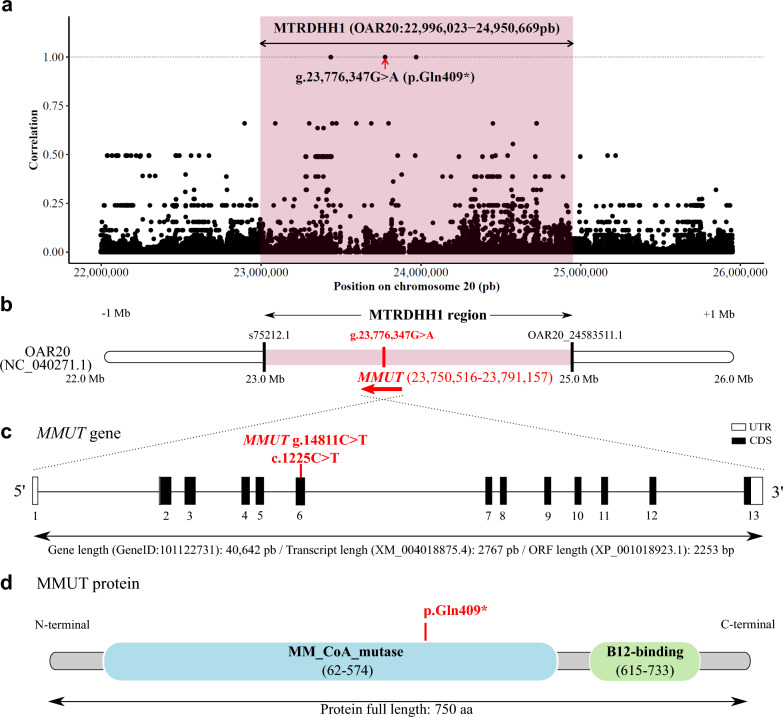
Nonsense variant in the *MMUT* gene within the MTRDHH1 genomic region. **a** Scatter plot showing the correlation between MTRDHH1 status (NC_040271.1, OAR20:22,996,023–24,950,669 extended from each side by 1 Mb) and genotype of variants from 100 whole-genome sequenced animals. Each dot represents one variant. **b** Position of the *MMUT* gene within the MTRDHH1 haplotype. Black bars indicate the first and the last markers of the Illumina Ovine SNP50 BeadChip defining the limits of MTRDHH1 [see Additional file 5: Table S4]. **c** *MMUT* gene structure (GeneID: 101,122,731) and localization of the *MMUT* C > T polymorphism identified in the sixth exon (XM_004018875.4, UTR: untranslated region; CDS: coding sequence). **d** MMUT protein (XP_004018923.1) with methylmalonyl-CoA mutase (PF01642) and B12-binding (PF02310) Pfam domain annotations (UniProtKB accession number: A0A6P3T7X3_SHEEP). The mutation creates a premature stop-gain at amino-acid position 409

Since the MTRDHH1 haplotype had the greatest impact on stillbirth rate that increased by + 7.5% in at-risk matings (Fig. 1), we focused on this haplotype, in particular, as it may represent a putative recessive lethal haplotype. In order to identify the MTRDHH1 causal mutation, we considered biallelic variants, SNP and insertion-deletions, for 100 ovine WGS among which 22 were from Manech Tête Rousse dairy sheep and two of them were heterozygous carriers of the MTRDHH1 haplotype. Within the MTRDHH1 region extended by 1 Mb on each side, 78,019 variants were called with a quality score > 30 and a call rate > 95%, and only four candidate variants had a perfect correlation (r^2^ = 1) between biallelic variant genotypes and the MTRDHH1 haplotype status (Additional file 8: Table S5 and Fig. 4a). Among these candidate variants, we identified two small insertions, one intergenic single nucleotide variant (SNV), and one nonsense (stop-gain) SNV located in the *MMUT* gene. This latter SNV (NC_040271.1: g.23,776,347G > A; XM_004018875.4: c.1225C > T; Fig. 4b, c) in the *MMUT* gene is predicted to create a premature stop codon at position 409 encoded by exon 6 (XP_004018923.1:p.Gln409*) whereas the full protein length is composed of 750 amino acids (Fig. 4d). The presence of this variant will disrupt the methylmalonic coenzyme-A mutase domain and result in the loss of the vitamin B12 binding domain.

**Fig. 5:**
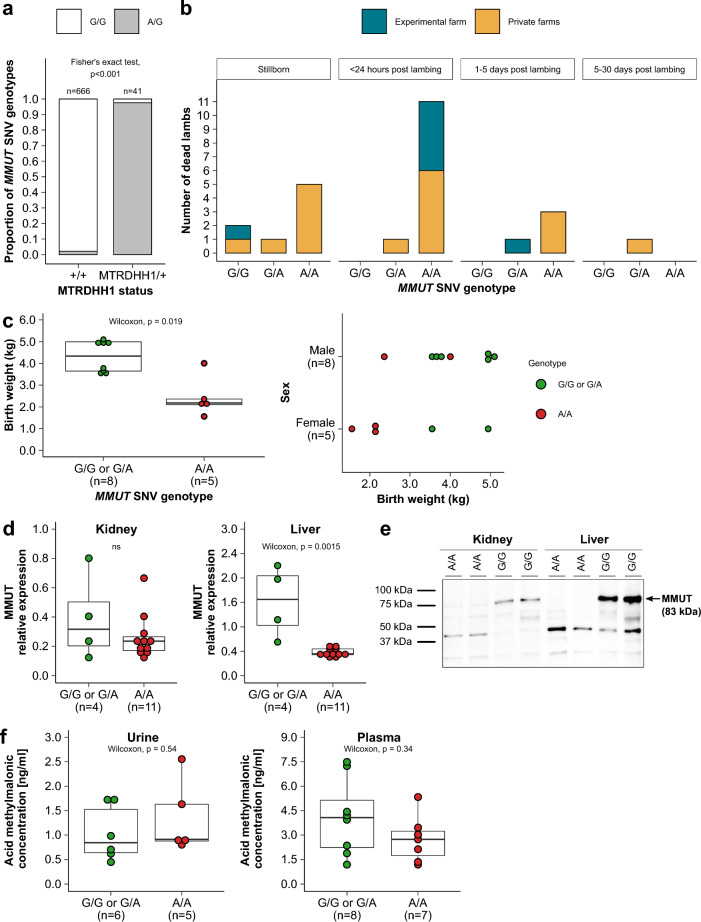
Effects of the *MMUT* nonsense variant. **a** Association of *MMUT* SNV genotypes with MTRDHH1 status. + / + : non-carriers; MTRDHH1/ + : heterozygous carriers and MTRDHH1/MTRDHH1: homozygous carriers (Fisher’s exact test, p < 0.001, without the homozygous MTRDHH1 carriers). **b** Time distribution of dead lambs in the pre-weaning period depending on *MMUT* SNV genotype and lambing place. **c** *MMUT* SNV genotype effect on lamb birth weight born on the experimental farm. Affected homozygous lambs are in red (A/A genotype). **d** *MMUT* gene expression at mRNA and **e** protein levels in kidney and liver depending on *MMUT* SNV genotypes. **f** Methylmalonic acid dosage by ELISA quantification in blood plasma and urine collected from lambs with different genotypes at the *MMUT* SNV

To validate the association between the *MMUT* variant and MTRDHH1 haplotype, we genotyped the cohort of male lambs born in 2021 (n = 714) with a genotyping test specific to *MMUT* g.23,776,347G > A SNV. The allele frequency of variant A was 3.8%. All these animals have a known status at the MTRDHH1 locus and the contingency table indicates a clear association between the MTRDHH1 haplotype status and *MMUT* variant genotypes (Fig. 5a, Fischer’s exact test p < 0.001). However, 15 animals showed discrepancy between *MMUT* and MTRDHH1 genotypes that are supposed to be in perfect linkage disequilibrium. A specific focus on the haplotypes carried by these animals in the MTRDHH1 region from marker 1 (s75212.1) to marker 32 (OAR20_24583511.1) showed that the 14 animals heterozygous for the variant exhibited shorter recombinant versions of the MTRDHH1 haplotype (see Additional file 9: Fig. S5). Nonetheless, one animal was heterozygous for the MTRDHH1 haplotype but did not carry the *MMUT* variant.

### Occurrence of the *MMUT* SNV in a sheep diversity panel

A sheep diversity panel composed of 25 French sheep breeds, including MTR, and three Latxa Spanish sheep breeds related to MTR was used to genotype the *MMUT* g.23,776,347G > A SNV (see Additional file 10: Table S6). As expected, some MTR animals (n = 5) from this panel were identified as heterozygous carriers, and the variant was also detected in one animal of the Spanish Latxa Cara Rubia population. None of the other animals tested carried the polymorphism.

### Viability of homozygous lambs for the *MMUT* variant

To validate the impact of the *MMUT* A variant allele, we organized at-risk mating between heterozygous carriers to generate homozygous lambs. Blood samples were collected from 181 MTR ewes, which were the daughters of MTRDHH1 haplotype carrier sires, on six private farms. The *MMUT* SNV specific genotyping identified 82 heterozygous ewes. Among these ewes, 73 were raised on the six private farms (Experiment 1) and nine were moved to an INRAE experimental farm (Experiment 2). All ewes were artificially inseminated with fresh semen from *MMUT* g.23776347G/A heterozygous rams. Forty-five days after AI, 42 ewes in Experiment 1 and five ewes in Experiment 2 were diagnosed as pregnant. This corresponds to an AIS of 59.8% in accordance with the average AIS of 60.9% determined previously in the whole population. In Experiment 1, only 37 among the 44 pregnant ewes were monitored after gestation diagnosis and resulted in the birth of 59 lambs (mean prolificacy of 1.6, litter size ranging from 1 to 3) with a gestation length between 139 and 159 days. In Experiment 2, the five pregnant ewes gave birth to 13 lambs (mean prolificacy of 2.6, litter size ranging from 2 to 4) with a gestation length between 151 and 157 days. No abortion during the five months of gestation was observed. Finally, 72 lambs (52% males and 48% females) were born and an ear punch was collected for genotyping of the *MMUT* SNV (Table 3). The distribution of genotypes did not differ between the two experiments (Fisher’s exact test, p = 0.5862). In total, 20 lambs were genotyped homozygous carriers (A/A), 30 heterozygous carriers (A/G) and 21 homozygous non-carriers (G/G). All lambs were monitored during the 0–30 day-period until weaning. Twenty-five lambs died during this period representing a huge mortality rate of 34.7% (Fig. 5b). Contingency table between lamb genotypes (A/A, G/A, G/G) and viability (alive or dead) indicated a higher mortality rate for homozygous A/A lambs (Table 3, Fisher’s exact test, p < 0.001). Indeed, the A/A dead lambs accounted for 76% of the whole lamb mortality. The intra-genotype mortality rate was very high in A/A lambs (95.0%) compared to A/G (13.3%) and G/G (9.5%) lambs. The death of A/A homozygous lambs occurred very soon after birth within the first 24 h. Clinical examination of the dead lambs did not reveal any specific symptoms. One homozygous A/A lamb passed the weaning age (around 4 weeks). In addition, in Experiment 2, the 13 lambs were weighed at birth (males: 4.0 ± 0.9 kg, females: 2.9 ± 1.4 kg) and A/A lambs had a significantly lower birth weight compared to the other genotypes, regardless of gender (Wilcoxon’s non parametric test, p = 0.019) (Fig. 5c).

**Table 3:** *MMUT* SNV genotyping results for lambs produced from at-risk matings on private and experimental farms

Experiment	G/G	G/A	A/A	−/−	Total
Private farms (n = 6)	17 (16ª/1^b^)	26 (23ª/3^†^)	15 (1ª/14^b^)	1* (0ª/1^b^)	59
Male	9	9	10	1	29
Female	8	17	4		29
Undetermined			1		1
Experimental farm (n = 1)	4 (3ª/1^b^)	4 (3ª/1^b^)	5(0ª/5^b^)		13
Male	4	2	2		8
Female	0	2	3		5
All	21 (19ª/2^b^)	30 (26ª/4^b^)	20 (1ª/19^b^)	1* (0ª/1^b^)	72
Male	13	11	12	1	37
Female	8	19	7	0	34
Undetermined			1		1

*The ear punch was not available for this lamb

ªNumber of alive lambs

^**b**^Number of dead lambs

### MMUT protein expression and activity

Based on the sheep gene atlas (http://biogps.org/sheepatlas/; accessed 17 February 2022), the *MMUT* gene is known to be highly expressed in the kidney and liver [27]. In order to investigate a putative nonsense-mediated mRNA decay (NMD) due to the nonsense variant in the *MMUT* gene, we evaluated the *MMUT* mRNA relative expression in kidney and liver by qPCR (Fig. 5d). We identified a significant reduction of *MMUT* expression in liver from A/A lambs (Wilcoxon’s non parametric test, p = 0.0015) but not in kidney (Wilcoxon’s non-parametric test, p = 0.66). We also assessed the MMUT protein expression from liver and kidney protein extracts collected from two homozygous A/A and two homozygous G/G dead lambs. As expected, using Western blotting, the wild type protein was expressed in kidney and liver whereas the mutated protein was not detected at least as a full-length form in both tissues (Fig. 5e). We also tried to evaluate the accumulation of MLA quantified by ELISA in the urine and blood of A/A lambs collected post-mortem or soon after birth in live animals (Fig. 5f) but no significant difference was observed compared to G/G or G/A lambs (Wilcoxon’s non-parametric test, p = 0.54 in urine, Wilcoxon’s non-parametric test, p = 0.34 in plasma).

## Discussion

Using a reverse genetic screen in the MTR population, we successfully identified five genomic regions (named MTRDHH1 to 5) with a significant deficit of homozygous animals, ranging from 84 to 100%. Compared to our previous analysis in Lacaune dairy sheep, which identified eight independent haplotypes, we identified fewer deficient haplotypes in MTR. This is possibly due to the smaller number of genotyped animals in MTR (19,102 Lacaune vs 6845 MTR [14]). In the MTR population, we estimated the frequencies of MTRDHH haplotype heterozygous carriers as ranging from 7.8 to 16.6%, and thus the allele frequency as ranging from 3.9 to 8.3%. This is in line with an allele frequency of 5%, which was expected from the analysis of a population of 6000 genotyped animals, as previously reported [28]. The current frequency of lethal variants in livestock is often the result of genetic drift, which is especially prominent in populations with small effective population sizes [9, 29]. However, many examples of balancing selection for deleterious alleles have also been described in livestock [30]. For example, in sheep, a missense variant in the *FGFR3* gene was found to be associated with enhanced skeletal growth and meat yield when in the heterozygous state, but to induce chondrodysplasia (spider lamb syndrome) when in the homozygous state (OMIA 001703-9940) [31]. Therefore, we sought to determine an impact of each MTRDHH haplotype on production traits to search for a selective advantage in the heterozygous state. The positive effects of these DHH haplotypes on selected traits were quite weak and only significant for MTRDHH2 haplotype carriers on milk yield and for MTRDHH5 haplotype carriers on fat yield. Thus, it is unlikely that selective advantage explains the observed DHH haplotype frequencies, or the rapid increase in MTRDHH3 haplotype frequency between 2017 and 2018 (Fig. 3). There was only a slight increase in the number of genotyped rams in 2018 (n = 591) compared to 2017 (n = 529). This upward trend for the MTRDHH3 haplotype could be attributed to genetic drift.

The populational analysis of AIS and SBR, recorded on more than 300,000 matings, allowed us to classify the different MTRDHH haplotypes based on their supposed deleterious impact on early gestation (AIS), around the time of birth (SBR), or on postnatal viability or morphological phenotypes when AIS and SBR were not altered. The MTRDHH1 haplotype on OAR20 was the only one with a strong negative impact exclusively on SBR, which suggests that this haplotype harbors a lethal variant affecting the perinatal period. Using WGS data from 22 MTR animals and including two MTRDHH1 haplotype heterozygous carriers, a single-nucleotide variant (SNV) at position g.23,776,347 on OAR20, appeared to be a strong functional candidate. This SNV, which is a G > A substitution on OAR20 (c.1225C > T on cDNA), leads to a nonsense mutation in the *MMUT* gene, which introduces a premature stop codon (p.Gln409*). Genotyping of g.23,776,347G > A in 714 animals with a known MTRDHH1 status showed an almost perfect association between the two. The only 15 discordant animals were largely explained by shorter recombinant versions of the MTRDHH1 haplotype (between 2 and 31 markers surrounding the SNV). Only one MTRDHH1 heterozygous carrier did not carry the *MMUT* variant. This discrepancy could be attributed to errors from SNP array genotyping, phasing and/or imputation, or recombination events on both sides of the SNV. Immunoblotting of liver and kidney protein extracts from homozygous carrier lambs confirmed the predicted impact of the candidate SNV on the protein. The anti-MMUT antibody detected a band at 83 kDa as expected for the full-length MMUT polypeptide in wild-type extracts. However, the MMUT band was not detected in homozygous carriers due to the lack of the antigenic epitope (from amino acid 451 to 750) in the p.Gln409* truncated form. This proves that the SNV has an effect on the functional expression of the *MMUT* gene, which is further supported by a nonsense-mediated decay phenomenon detected in liver. To confirm the perinatal lethality of the MTRDHH1 haplotype, which was associated with a 7.5% increase in SBR, we conducted at-risk matings between heterozygous carriers of the p.Gln409* variant. In this experiment, 84% of the homozygous lambs died within the first 24 h after birth, in line with the hypothesis. This also explains the deficit of the homozygous carriers of the MTRDHH1 haplotype in the DHH analysis, as they would have died before they could be genotyped. In spite of the observed perinatal lethality in homozygous carrier lambs, we did identify one homozygous lamb that survived weaning. This individual was subsequently slaughtered for commercial purposes at approximately one month of age, preventing further data collection. While this finding raises the question of whether the mutation is causal, this variant is the most likely functional candidate within the MTRDHH1 region. In vitro analyses further support this possibility, demonstrating impaired MMUT expression with a truncated protein. The more plausible explanation is that the mutation exhibits incomplete penetrance, meaning that not all individuals that carry the mutation develop the associated phenotype. For example, homozygous knock-out mice for *Mmut* (MGI:97239) exhibited neonatal lethality, but a few animals survived beyond weaning exhibiting postnatal growth retardation [32]. In cattle, a deleterious mutation in the *CAD* gene (p.Tyr452Cys, NH7 haplotype in Normande) also caused embryonic lethality with incomplete penetrance, with one documented surviving homozygous cow [33].

We also showed that homozygous newborn lambs had a lower birth weight, an observation which could be compared to the *Mmut* knock-out mice as described above (MGI:97239). In humans, numerous pathogenic variants of the *MMUT* gene cause “methylmalonic aciduria” (OMIM 609058, MMA, [34]), an autosomal recessive metabolism disorder. MMUT is part of a metabolic pathway starting from the degradation of amino acids (valine, isoleucine, methionine and threonine), odd-chain fatty acids, cholesterol and propionic acid to succinyl-CoA by three main enzymes: propionyl-CoA carboxylase (PCC), methylmalonyl-CoA epimerase (MCE) and methylmalonyl-CoA mutase (MMUT) [35, 36]. The MMUT protein is a mitochondrial enzyme that catalyzes the L-methylmalonyl-CoA to succinyl-CoA, an intermediate in the Krebs cycle. The isomerization of methylmalonyl-CoA requires adenosylcobalamin (AdoCbl), the cofactor form of vitamin B12 (also known as cobalamin) [37]. In sheep, the mutant protein (p.Gln409*) does not carry the B12 binding domain (615–733 amino acids), suggesting that the AdoCbl cofactor is unable to act in the conversion of L-methylmalonyl-CoA to succinyl-CoA. When the MMUT enzyme is not functional, MLA accumulates in body fluids, mainly in the blood and urine [36, 38]. However, we failed in evidencing such MLA accumulation in the plasma or urine of homozygous A/A lambs, while this was observed in homozygous knock-out mice 24 h after birth [39]. In our study, many of our urine and blood samples were collected at necropsy only after natural death of the lambs without time control, possibly affecting the results. The methylmalonic aciduria in this sheep genetic model will need further clinical investigation.

The evidence presented in this study clearly indicates that the deficit of homozygous carriers of the MTRDHH1 haplotype is due to a recessive loss-of-function mutation in the *MMUT* gene that alters an essential metabolic pathway. This is consistent with the findings of many other studies that have shown an association between DHH and mutations in genes involved in metabolism [9]. In bovine, many variants have been shown to affect metabolic processes in several breeds, including Braunvieh (BH24/*CPT1C,* lipid metabolism) [40], Holstein (HH4/*GART*, nucleotide metabolism) [41], Montbéliarde (MH1/*PFAS,* nucleotide metabolism; MH2/*SLC37A2,* glucose metabolism) [41, 42], Normande (NH7/*CAD,* nucleotide metabolism) [33], and Simmental (SH8/*CYP2B6*, respiratory chain) [43].

The diversity analysis also revealed the segregation of the *MMUT* SNV in the Spanish Latxa Cara Rubia (LCR) breed. The French MTR and Spanish LCR are very closely related populations. Since the 1970s, there have been many exchanges between these populations across the border, first from Spain to France during the 1970s, and then the reverse since the 1990s [44]. The *MMUT* SNV discovered in this study is not shared by other French sheep breeds, or by the individuals from the International Sheep Genome Consortium (ISGC, a dataset composed of 453 animals from 38 breeds all over the world, https://www.sheephapmap.org/), or from the Flock54℠ program (5061 sheep, https://www.flock54.com) [45]. However, searching the ISGC dataset, we found another SNV located in the ovine *MMUT* gene (rs1093255812) that leads to a premature stop-gain (ENSOART00020022357.1: p.Trp43*). This variant was identified in the heterozygous state in the New Zealand Coopworth breed, and as in MTR, it could have the same deleterious impact on lamb viability.

This study identified a lethal variant in the *MMUT* gene, which is harbored by the MTRDHH1 haplotype. Four other putative lethal or deleterious variants are still to be identified in other homozygous deficient haplotypes. Based on the ovine gene annotation of the MTRDHH2 (OAR1), MTRDHH3 (OAR1), MTRDHH4 (OAR10) and MTRDHH5 (OAR13) regions, we searched the MGI, IMPC, OMIM and OMIA databases for genes involved in lethal phenotypes, inherited disorders and morphological traits (see Additional file 11: Table S7). For the MTRDHH2 haplotype, which is associated with significant negative effects on AIS and SBR, we hypothesized that the causative variant could induce embryo/fetal losses throughout the gestation period and until birth. In this region, only the *SLC33A1* gene has been shown to impact both embryonic lethality and survival rate when knocked-out in mice (MGI:1332247). Therefore, *SLC33A1* appears to be an obvious candidate gene. In contrast, the MTRDHH3, MTRDHH4, and MTRDHH5 haplotypes did not have a significant impact on fertility traits. We hypothesize that these haplotypes harbor deleterious variants that lead to postnatal lethality or are associated with morphological defects that are selected against at the time of candidate lamb genotyping. Candidate genes associated with postnatal lethality are mainly involved in metabolism (*TARS2* in the MTRDHH3 region, *KL* in the MTRDHH4 region) or DNA repair (*BRCA2* in the MTRDHH4 region). Other genes, which are not necessarily associated with lethality, may affect animals with vision alterations (*PRPF3*, *ADAMTSL4* in the MTRDHH3 region, and *KIF3B* in the MTRDHH5 region), neurological disorders (*PRUNE1*, *POGZ* in the MTRDHH3, and *ASXL1*, *PIGU*, *AHCY* in the MTRDHH5 region), or morphological/stature defects (*ITGA10*, *POLR3GL*, *ECM1* in the MTRDHH2 region, *RXFP2* in the MTRDHH4 region, and *DNMT3B*, *CEP250*, *ASIP* in the MTRDHH5 region). The presence of *RXFP2* (MTRDHH4 region) and *ASIP* (MTRDHH5 region) as positional candidate genes is puzzling since these genes are already known to harbor variants that control the horned/polled phenotype (OMIA 000483-9940) [46] and black coat color (OMIA 000201-9940) [47, 48] in sheep, respectively. Interestingly, in the MTR selection scheme, horned females and black animals do not fit with breed standards and are therefore not desirable. This may lead to the exclusion of these animals from genotyping, which fully express their phenotype (horn or black coat) in the homozygous state.

## Conclusions

In this study, first we identified in the MTR dairy sheep the segregation of five independent haplotypes possibly harboring five recessive deleterious alleles of which at least two have a negative impact on fertility traits. Among these, we detected the *MMUT* c.1225T (p.Gln409*) variant associated with the MTRDHH1 haplotype causing early lamb mortality when in the homozygous state. Further clinical studies are warranted to evaluate whether the methylmalonic aciduria syndrome occurs in sheep as in humans with the same recessive genetic determinism on *MMUT*. MTRDHH2 represents a promising haplotype to identify another recessive lethal mutation in the near future. Anyway, specific management of these haplotypes/variants in the MTR dairy sheep selection program would help enhance the overall fertility and lamb survival.

### Supplementary Information

**Additional file 1: Figure S1.** Distribution of genotyped animals over time. The bar charts represent the number of genotyped animals according to sex and year of birth. The genomic selection in MTR dairy sheep was implemented in 2017.

**Additional file 2:** **Table S1.** EMBL-EBI accession numbers of the 100 whole-genome sequences used in the analysis [15, 26, 49–51].

**Additional file 3: Table S2. **List of PCR primer sequences.

**Additional file 4: Figure S2**. Experimental design to generate MMUT homozygous variant lambs.

**Additional file 5:** **Table S3**. Clustering 20-SNP haplotypes into MTRDHH regions. The table shows all significant haplotypes of 20 markers (20-SNP haplotypes with frequency > 1%, P-value < 1.9 × 10^−4^ and deficit ≥ 75%). As described in the Methods section, some of the consecutive 20-SNP haplotypes could be clustered into five MTRDHH regions based on similar parameters. **Table S4**. SNPs defining the MTRDHH regions. The table gives the position of each SNP within the MTRDHH regions according to the sheep reference genomes Oar_v3.1, Oar_rambouillet_v1.0 and ARS-UI_Ramb_v2.0, and the phased alleles of each deficient haplotype.

**Additional file 6: Figure S3.** Manhattan plot of 20-SNP haplotypes in Manech Tête Rousse dairy sheep. Each point represents a haplotype of 20 markers with a frequency higher than 1% in the maternal phase. The red line represents the P-value threshold (1.9 × 10^−4^), which was used to identify haplotypes with a significant deficit in homozygotes. Only 20-SNP haplotypes with a deficit in homozygotes of at least 75% (green dots) were selected, resulting in the identification of 150 significant 20-SNP haplotypes clustered in five regions (MTRDHH1 to 5). Genomic coordinates refer to the sheep reference genome Oar_v3.1.

**Additional file 7: Figure S4**. Total merit genomic index (ISOLg) of the 2021 MTR genomic cohort lambs (n = 714). (**a**) Distribution of ISOLg, determined by a combination of four selected traits: MY, FC, PC and LSCS. Comparison of ISOLg according to DHH status, (**b**) MTRDHH1, (**c**) MTRDHH2, (**d**) MTRDHH3, (**e**) MTRDHH4, (**f**) MTRDHH5.

**Additional file 8: Table S5.** Candidate variants located in the MTRDHH1 region.

**Additional file 9: Figure S5**. MTRDHH1 recombinant haplotypes from 15 animals with discrepancy between MTRDHH1 status and *MMUT* SNV genotype. MTRDHH1/ + and + / + refer to heterozygous and non-carriers of MTRDHH1, respectively. The grey column represents the localization of the *MMUT* SNV (g.23,776,347G > A) within the MTRDHH1 haplotype. For each animal, only the phase supposed to harbour the *MMUT* SNV variant allele A is represented. The blue colour indicates the portion of local haplotypes matching with the MTRDHH1 haplotype.

**Additional file 10: Table S6.** *MMUT* SNV genotype distribution from a DNA diversity panel of French and Spanish ovine breeds.

**Additional file 11: Table S7**. List of the 408 protein coding genes located in the five MTRDHH regions extended by 1 Mb on each side. Mouse knock-out (KO) phenotypes and association with mammalian disorders were extracted for each gene from several databases: MGI: www.informatics.jax.org; IMPC: https://www.mousephenotype.org; OMIM: Online Mendelian Inheritance in Man (https://omim.org) and OMIA: Online Mendelian Inheritance in Animal (https://omia.org). For mouse KO phenotypes associated with lethality, the affected developmental stages is indicated by 1 (0, no lethality).

## Data Availability

The SNP chip genotyping data that support the findings of this study are available from the official French livestock data system (Systèmes Nationaux d’Information Génétique, France Génétique Elevage, Paris, France), but restrictions apply to the availability of these data, which were used under license for the current study, and so are not publicly available. Data are however available from the authors upon reasonable request and with permission of CDEO, CNBL, FGE and Valogène. The whole-genome sequence data used in this study are publicly available, EMBL-EBI accession numbers are described in Additional file 2: Table S1.
