# Supplementary figures and images for: Searching for homozygous haplotype deficiency in Manech Tête Rousse dairy sheep revealed a nonsense variant in the MMUT gene affecting newborn lamb viability
Source: Genet Sel Evol. 2024 Feb 29;56:16. doi: 10.1186/s12711-024-00886-7 (PMC10905913; doi:10.1186/s12711-024-00886-7)

Number of genotyped animals

sex

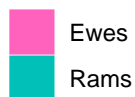

Implementation of  
genomic selection

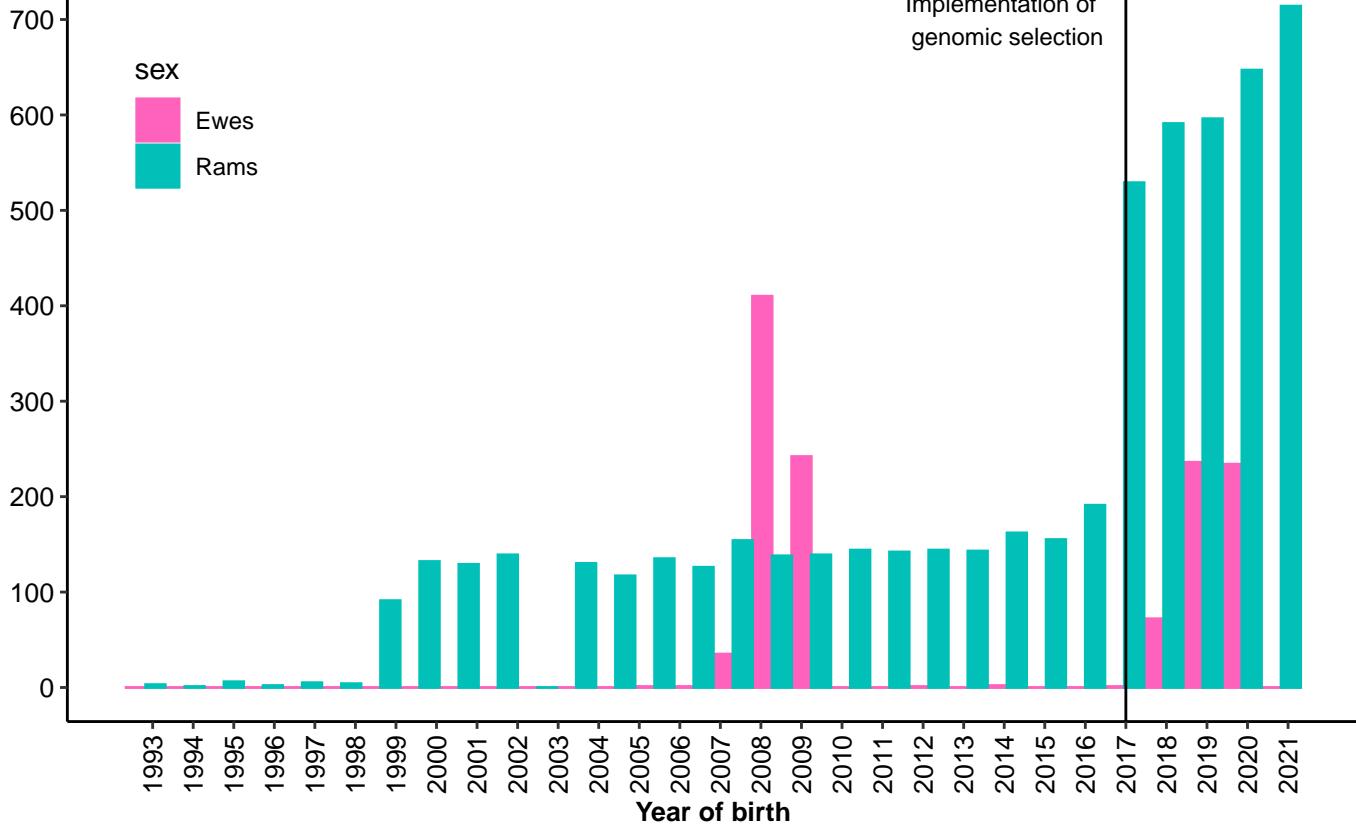

Supplement: Supplementary file 1 — Additional file 1: Figure S1. Distribution of genotyped animals over time. The bar charts represent the number of genotyped animals according to sex and year of birth. The genomic selection in MTR dairy sheep was implemented in 2017. [file 12711_2024_886_MOESM1_ESM.pdf]

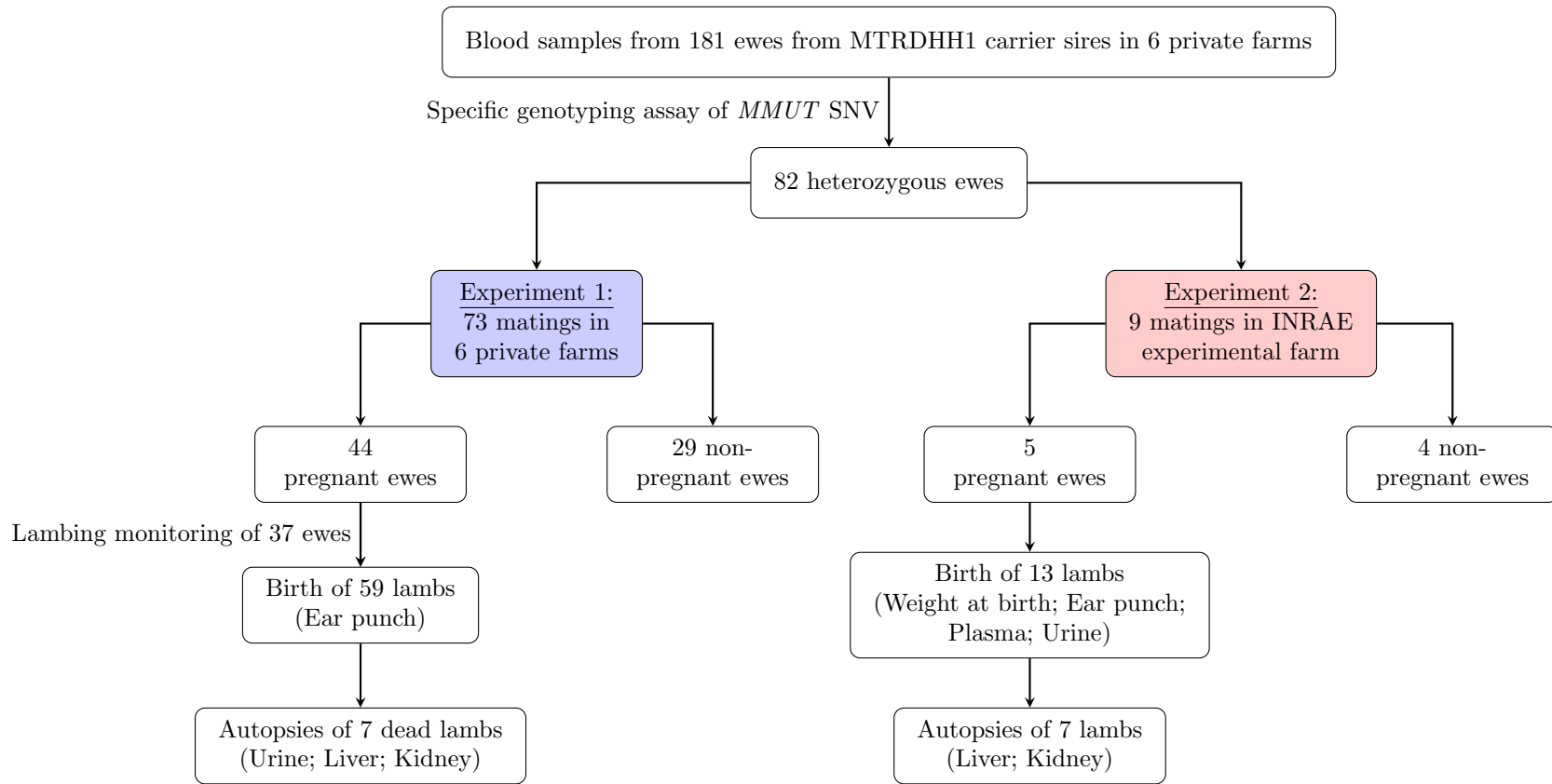

Supplement: Supplementary file 4 — Additional file 4: Figure S2. Experimental design to generate MMUT homozygous variant lambs. [file 12711_2024_886_MOESM4_ESM.pdf]

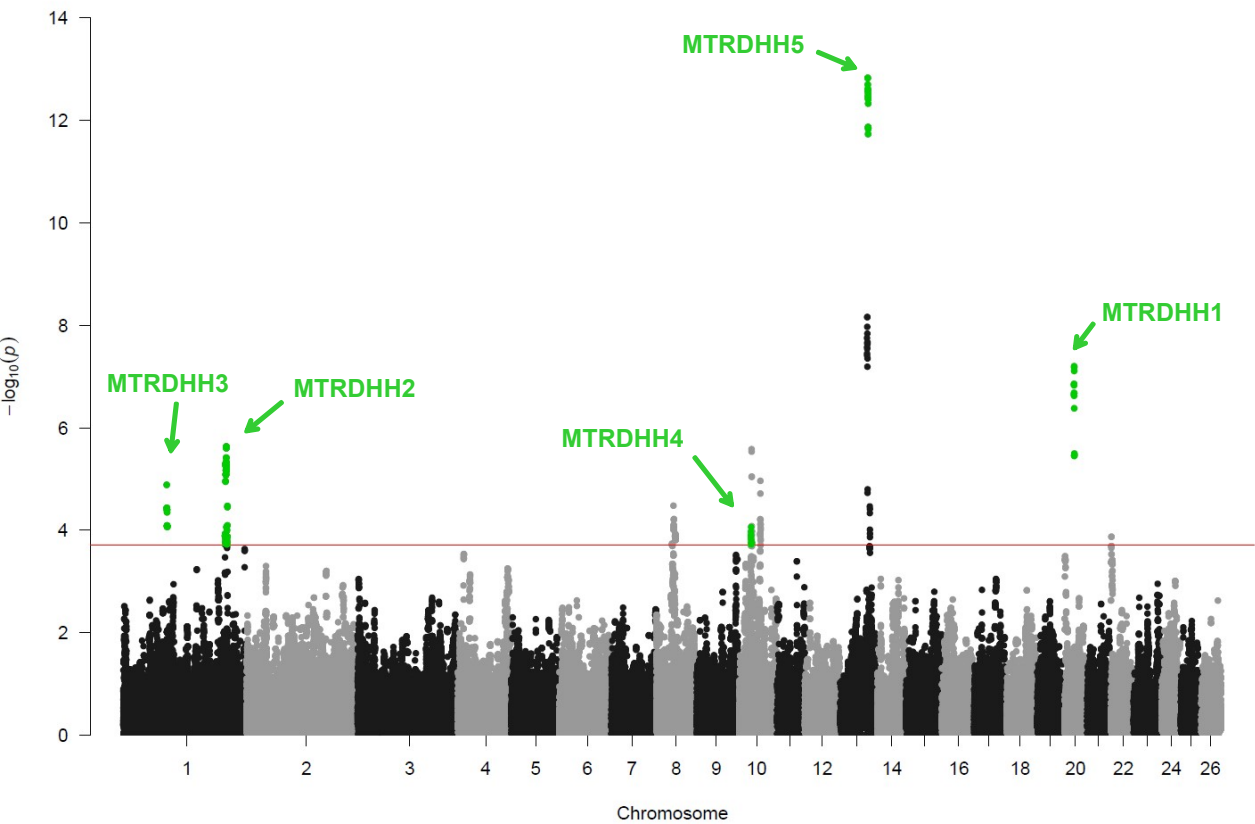

Supplement: Supplementary file 6 — Additional file 6: Figure S3. Manhattan plot of 20-SNP haplotypes in Manech Tête Rousse dairy sheep. Each point represents a haplotype of 20 markers with a frequency higher than 1% in the maternal phase. The red line represents the P-value threshold (1.9 × 10^−4^), which was used to identify haplotypes with a significant deficit in homozygotes. Only 20-SNP haplotypes with a deficit in homozygotes of at least 75% (green dots) were selected, resulting in the identification of 150 significant 20-SNP haplotypes clustered in five regions (MTRDHH1 to 5). Genomic coordinates refer to the sheep reference genome Oar_v3.1. [file 12711_2024_886_MOESM6_ESM.pdf]

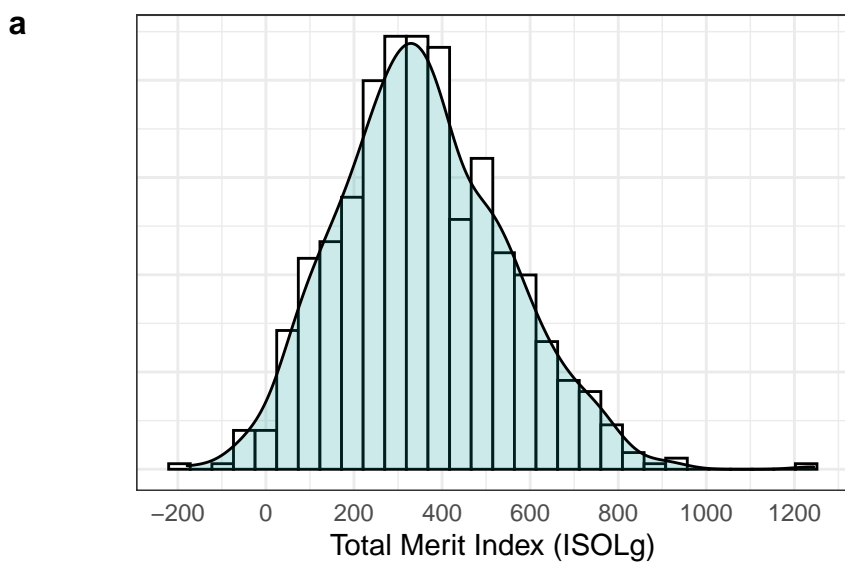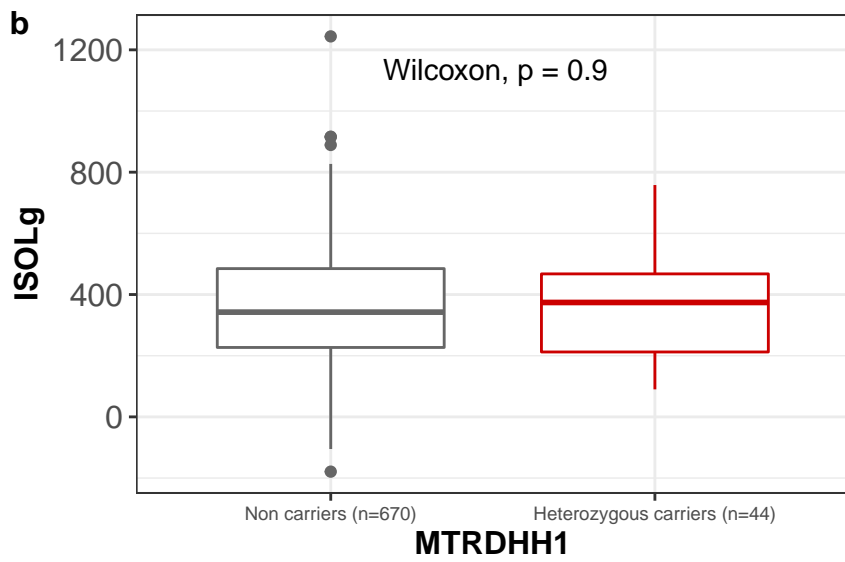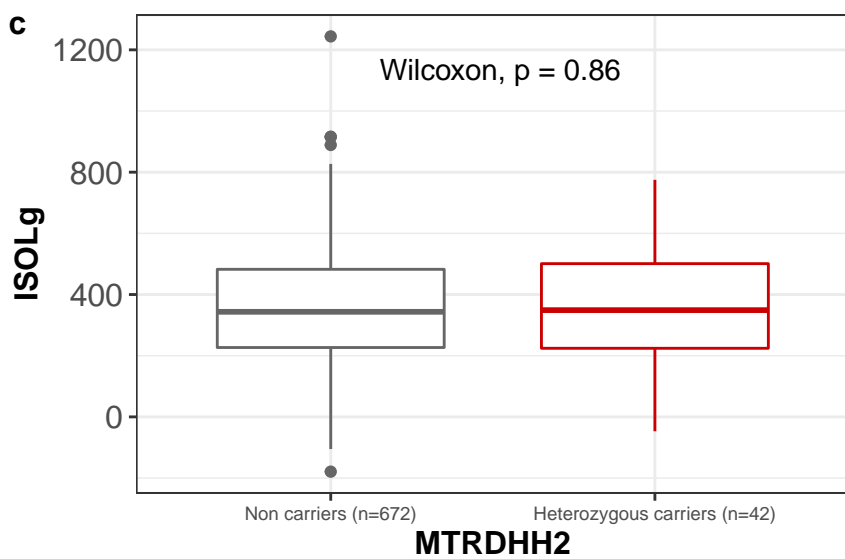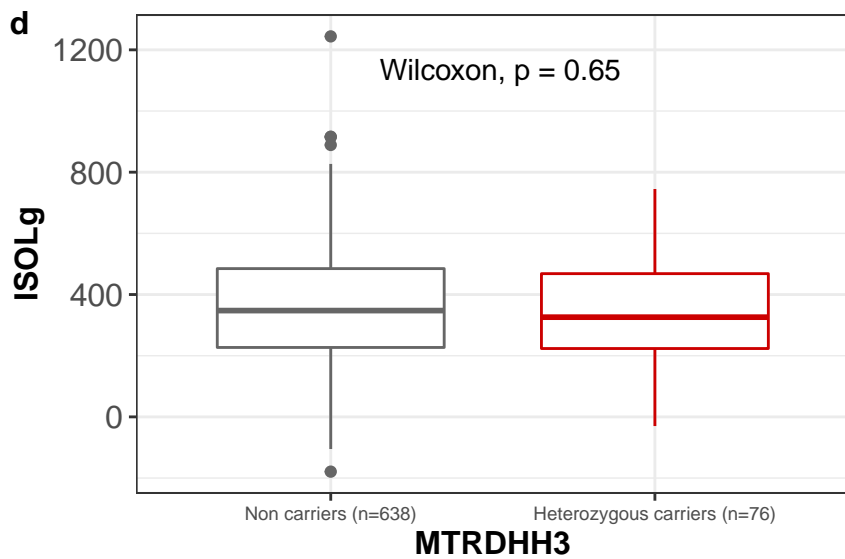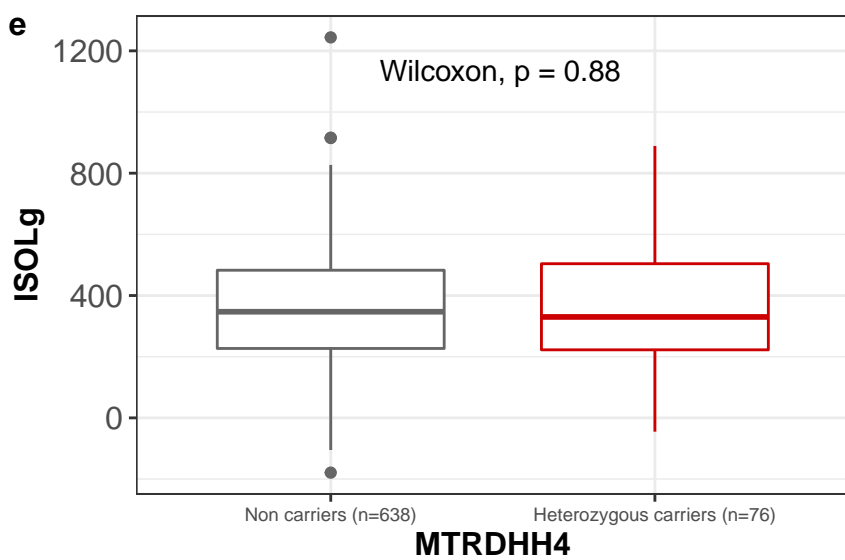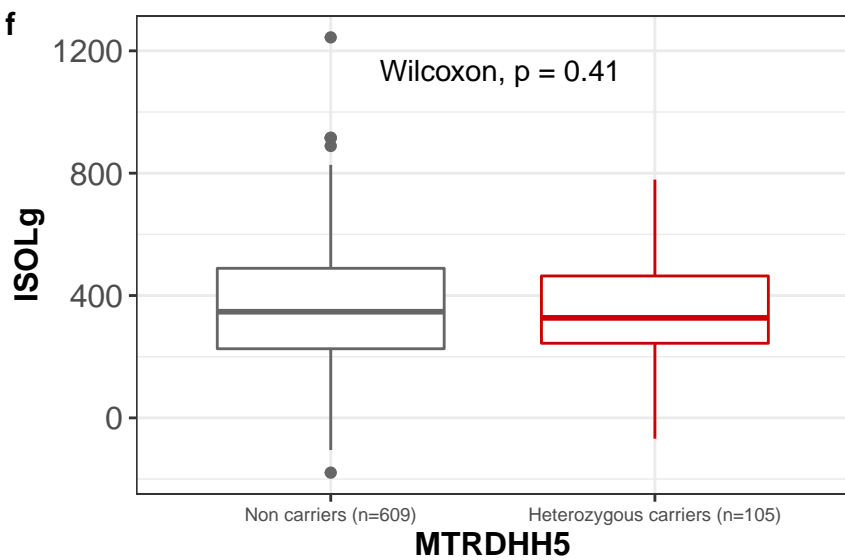

Supplement: Supplementary file 7 — Additional file 7: Figure S4. Total merit genomic index (ISOLg) of the 2021 MTR genomic cohort lambs (n = 714). (a) Distribution of ISOLg, determined by a combination of four selected traits: MY, FC, PC and LSCS. Comparison of ISOLg according to DHH status, (b) MTRDHH1, (c) MTRDHH2, (d) MTRDHH3, (e) MTRDHH4, (f) MTRDHH5. [file 12711_2024_886_MOESM7_ESM.pdf]
