# Supplementary material for: Searching for homozygous haplotype deficiency in Manech Tête Rousse dairy sheep revealed a nonsense variant in the MMUT gene affecting newborn lamb viability
Source: Genet Sel Evol. 2024 Feb 29;56:16. doi: 10.1186/s12711-024-00886-7 (PMC10905913; doi:10.1186/s12711-024-00886-7)
Supplement: Supplementary file 2 — Additional file 2: Table S1. EMBL-EBI accession numbers of the 100 whole-genome sequences used in the analysis [15, 26, 49–51]. [file 12711_2024_886_MOESM2_ESM.pdf]

Additional file 2: Table S1. EMBL-EBI accession numbers of the 100 whole-genome sequences used in the analysis.

Breed	Number of animals	ENA run accession	EBI project accession	Associated references
Belclare	2	SRR14934360; SRR14935129	PRJNA698548	
Berrichon du Cher	3	ERS1205899; ERS1205900; ERS1205901	PRJEB14418	[49]
Cambridge	7	ERR1419201; ERR1419202; ERR1419203; ERR1419204; ERR1419205; ERR1419206; ERR1419207	PRJEB14098	
Charollais	1	SRR14934359	PRJNA698548	
Lacaune (Dairy)	31	ERR3276357; ERR3276358; ERR3276359; ERR3276360; ERR3276361; ERR3276362; ERR3276363; ERR3276364; ERR3276365; ERR3276366; ERR3276367; ERR3276368; ERR3276369; ERR3276370; ERR3276371; ERR3276372; ERR3276373; ERR3276374; ERR3276375; ERR3276376; ERR3276377; ERR3276378; ERR3276379; ERR7891349; ERR7891350; ERR7891351<br>ERR968423; ERR968424; ERR968425<br>SRR501850; SRR501851	PRJEB32110<br>PRJEB9911<br>PRJNA160933	[15,50]
Lacaune (Meat)	3	ERR3276380; ERR3276381; ERR3276382	PRJEB32110	
Manech Tête Rousse	22	ERR3712282; ERR3712283; ERR3712284; ERR3712285*; ERR3712286; ERR3712287; ERR3712288; ERR3712289; ERR3712290; ERR3712291; ERR3712292; ERR3712293; ERR3712294; ERR3712295; ERR3712296; ERR3712297; ERR3712298; ERR3712299; ERR3712300; ERR3712301*; ERR7889920; ERR7889921	PRJEB35682	
Martinik Blackbelly	1	ERR3255914	PRJEB31930	
Noire du velay	2	ERR3828659; ERR3828660	PRJEB35553	[26]
Romane	4	ERS1205902; ERS1205903; ERR2818429; ERR2818430	PRJEB14418	[49]
Romane x Martinik Blackbelly	13	ERR3255915; ERR3255916; ERR3255917; ERR3255918; ERR3255919; ERR3988554; ERR3988555; ERR3988556; ERR3988557; ERR3988558; ERR3988559; ERR3988560; ERR3988561	PRJEB31930	
Romanov	2	ERS1205904; ERS1205905	PRJEB14418	[49]
Suffolk	2	SRR14934357; SRR14934358	PRJNA698548	
Texel	2	SRR14934355; SRR14934356	PRJNA698548	
Vendéen	5	ERR4236129; ERR4236130; ERR4236131<br>SRR14934353; SRR14934354	PRJEB37460<br>PRJNA698548	[51]
**Total**	**100**			

* MTRDHH1 heterozygous carrier
