# Supplementary material for: Searching for homozygous haplotype deficiency in Manech Tête Rousse dairy sheep revealed a nonsense variant in the MMUT gene affecting newborn lamb viability
Source: Genet Sel Evol. 2024 Feb 29;56:16. doi: 10.1186/s12711-024-00886-7 (PMC10905913; doi:10.1186/s12711-024-00886-7)
Supplement: Supplementary file 3 — Additional file 3: Table S2. List of PCR primer sequences. [file 12711_2024_886_MOESM3_ESM.pdf]

Additional file 3: Table S2. List of PCR primer sequences

**Application**	**Gene symbol**	**Sequence (5'→3')**	**fragment size (pb)**	**Efficiency**
Terra PCR	*MMUT*<br>NC_040271.1	**F:** GCAGATTTTGGCAAAGAAAG<br>**R:** ACCTTCAAGGCAGCATCATA	402	-
PACE PCR	*MMUT*<br>NC_040271.1	**F1:** GAAGGTGACCAAGTTCATGCTAATCCCAGATTCTTCTTGAATAATGATTTG<br>**F2:** GAAGGTCGGAGTCAACGGATTGAATCCCAGATTCTTCTTGAATAATGATTTA<br>**R:** GTGAAAAGTGCTCGAATTGCCAGGAA	81/82	-
Quantitative PCR	*MMUT*<br>*XM_004018875*	**F:** GCTCATCAAGGAACTCAA<br>**R:** TTCAATATCATCAAGCACTTG	169	1.80
	GAPDH<br>NM_001190390	**F:** CGACTTCAACAGCGACACTC<br>**R:** TGCTGTAGCCGAATTCATTG	113	1.90
	YWHAZ<br>NM_001267887	**F:** ATTAAGTGAAGAGTCATACAA<br>**R:** GTATCCGATGTCCACAAT	81	2.00
	RPL19<br>XM_004012836	**F:** AATGCCAATGCCAACTC<br>**R:** CCCTTTCGCTACCTATACC	149	2.00
	SDHA<br>XM_027980212	**F:** GAATGGTCTGGAACACTG<br>**R:** AGTAATCGTACTCGTCAAC	156	2.00

F: Forward primer, **R:** Reverse primer.
