# Supplementary material for: Searching for homozygous haplotype deficiency in Manech Tête Rousse dairy sheep revealed a nonsense variant in the MMUT gene affecting newborn lamb viability
Source: Genet Sel Evol. 2024 Feb 29;56:16. doi: 10.1186/s12711-024-00886-7 (PMC10905913; doi:10.1186/s12711-024-00886-7)
Supplement: Supplementary file 8 — Additional file 8: Table S5. Candidate variants located in the MTRDHH1 region. [file 12711_2024_886_MOESM8_ESM.pdf]

Additional file 8 Table S5. Candidate variants located in MTRDHH1

Position	Ref/Alt	Quality score	Location Annotation	Functional Consequence[a]
23,436,234	G/GTCACA	385.8	Intergenic	Modifier
23,436,236	T/TTTGTG	385.8	Intergenic	Modifier
23,776,347	G/A	146.0	Exonic, *MMUT* (c.1225C>T)	High, stop-gain (p.Gln409*)
23,969,676	C/T	370.3	Intergenic	Modifier

[a]Variant annotation and effect predicted by SnpEff [23].
Ref: reference allele
Alt: alternate allele
