# Supplementary material for: Searching for homozygous haplotype deficiency in Manech Tête Rousse dairy sheep revealed a nonsense variant in the MMUT gene affecting newborn lamb viability
Source: Genet Sel Evol. 2024 Feb 29;56:16. doi: 10.1186/s12711-024-00886-7 (PMC10905913; doi:10.1186/s12711-024-00886-7)
Supplement: Supplementary file 10 — Additional file 10: Table S6. MMUT SNV genotype distribution from a DNA diversity panel of French and Spanish ovine breeds. [file 12711_2024_886_MOESM10_ESM.pdf]

Additional file 10: Table S6. ***MMUT*** **SNV genotype distribution from a DNA diversity panel of French and Spanish ovine breeds**

Breed	Total	G/G	A/G	Breed	Total	G/G	A/G
Berrichon du Cher (FR)	30	30		Martinik (FR)	23	23	
Blanche du Massif Central (FR)	31	31		Merinos d'Arles (FR)	27	27	
Causse du Lot (FR)	32	32		Mourerous (FR)	26	26	
Charmoise (FR)	31	31		Mouton Vendéen (FR)	30	30	
Charollais (FR)	30	30		Noir du Velay (FR)	28	28	
Corse (FR)	30	30		Préalpes du sud (FR)	27	27	
Ile de France (FR)	28	28		Rava (FR)	29	29	
Lacaune (Meat) (FR)	45	45		Romane (FR)	30	30	
Lacaune (Milk) (FR)	40	40		Romanov (FR)	26	26	
Latxa Cara Negra Euskadi (ES)	30	30		Rouge de l'Ouest (FR)	30	30	
Latxa Cara Negra Navarra (ES)	40	40		Roussin (FR)	30	30	
Latxa Cara Rubia (ES)	30	29	1	Suffolk (FR)	29	29	
Limousine (FR)	30	30		Tarasconnaise (FR)	33	33	
Manech Tête Rousse (FR)	29	24	5	Texel (FR)	27	27	
				Total	*851*	*845*	*6*

FR: France; ES: Spain
