# Supplementary material for: Searching for homozygous haplotype deficiency in Manech Tête Rousse dairy sheep revealed a nonsense variant in the MMUT gene affecting newborn lamb viability
Source: Genet Sel Evol. 2024 Feb 29;56:16. doi: 10.1186/s12711-024-00886-7 (PMC10905913; doi:10.1186/s12711-024-00886-7)
Supplement: Supplementary file 9 — Additional file 9: Figure S5. MTRDHH1 recombinant haplotypes from 15 animals with discrepancy between MTRDHH1 status and MMUT SNV genotype. MTRDHH1/ + and + / + refer to heterozygous and non-carriers of MTRDHH1, respectively. The grey column represents the localization of the MMUT SNV (g.23,776,347G > A) within the MTRDHH1 haplotype. For each animal, only the phase supposed to harbour the MMUT SNV variant allele A is represented. The blue colour indicates the portion of local haplotypes matching with the MTRDHH1 haplotype. [file 12711_2024_886_MOESM9_ESM.pdf]

MTRDHH1 haplotype
(32 markers)

Marker	Haplotype allele	SNP
SNP1	G	s75212.1 [A/G]
SNP2	G	OAR20_22661712_X.1 [A/G]
SNP3	G	OAR20_22784700.1 [A/G]
SNP4	A	OAR20_22851804.1 [A/C]
SNP5	G	s39376.1 [G/A]
SNP6	G	OAR20_22932011_X.1 [A/G]
SNP7	G	OAR20_22996638.1 [G/A]
SNP8	G	OAR20_23028179.1 [G/A]
SNP9	A	OAR20_23063796.1 [A/C]
SNP10	A	OAR20_23099257_X.1 [G/A]
SNP11	A	OAR20_23116747.1 [A/G]
SNP12	G	s66452.1 [A/G]
SNP13	G	OAR20_23240800.1 [A/G]
		***MMUT* SNV (G>A)**
SNP14	G	s04959.1 [G/A]
SNP15	A	s12178.1 [A/G]
SNP16	A	OAR20_23408992.1 [A/C]
SNP17	G	OAR20_23455049.1 [A/G]
SNP18	A	OAR20_23481589_X.1 [G/A]
SNP19	A	OAR20_23552034_X.1 [A/G]
SNP20	G	OAR20_23606688.1 [A/G]
SNP21	C	OAR20_23662826.1 [A/C]
SNP22	A	OAR20_23756997.1 [A/G]
SNP23	G	OAR20_23854908.1 [G/A]
SNP24	G	OAR20_23878963.1 [A/G]
SNP25	A	OAR20_23898925.1 [A/G]
SNP26	G	OAR20_24029500.1 [G/A]
SNP27	C	OAR20_24071930.1 [A/C]
SNP28	G	OAR20_24089242_X.1 [G/A]
SNP29	A	OAR20_24307087.1 [G/A]
SNP30	G	OAR20_24357620.1 [A/G]
SNP31	G	OAR20_24553232.1 [G/A]
SNP32	A	OAR20_24583511.1 [A/G]

Animal	MTRDHH1 status	*MMUT* genotype	*MMUT* SNV (G>A)
Animal 1	+/+	G/A	A
Animal 2	+/+	G/A	A
Animal 3	+/+	G/A	A
Animal 4	+/+	G/A	A
Animal 5	+/+	G/A	A
Animal 6	+/+	G/A	A
Animal 7	+/+	G/A	A
Animal 8	+/+	G/A	A
Animal 9	+/+	G/A	A
Animal 10	+/+	G/A	A
Animal 11	+/+	G/A	A
Animal 12	+/+	G/A	A
Animal 13	+/+	G/A	A
Animal 14	+/+	G/A	A
Animal 15	MTRDHH1/+	G/G	G
